# Working memory deficits in high-functioning adolescents with autism spectrum disorders: neuropsychological and neuroimaging correlates

**DOI:** 10.1186/1866-1955-5-14

**Published:** 2013-06-04

**Authors:** Evelien M Barendse, Marc PH Hendriks, Jacobus FA Jansen, Walter H Backes, Paul AM Hofman, Geert Thoonen, Roy PC Kessels, Albert P Aldenkamp

**Affiliations:** 1 Department for Research and Development, Kempenhaeghe, Expertise Centre for Epileptology, Sleep Medicine and Neurocognition, PO Box 61, 5590 AB, Heeze, The Netherlands; 2 Department of Neurology and School for Mental Health and Neuroscience (MheNS), Maastricht University Medical Centre, Maastricht, The Netherlands; 3 Department of Radiology, Maastricht University Medical Center, PO Box 5800, 6202 AZ, Maastricht, The Netherlands; 4 Special Education School de Berkenschutse, Sterkselseweg 65, 5591 VE, Heeze, The Netherlands; 5 Donders Institute for Brain, Cognition and Behaviour, Radboud University Nijmegen, PO Box 9104, 6500 HE, Nijmegen, The Netherlands; 6 Department of Medical Psychology, Radboud University Nijmegen Medical Centre, Nijmegen, The Netherlands

**Keywords:** Working memory, Adolescents, Autism, Neuropsychology, Neuroimaging

## Abstract

Working memory is a temporary storage system under attentional control. It is believed to play a central role in online processing of complex cognitive information and may also play a role in social cognition and interpersonal interactions. Adolescents with a disorder on the autism spectrum display problems in precisely these domains. Social impairments, communication difficulties, and repetitive interests and activities are core domains of autism spectrum disorders (ASD), and executive function problems are often seen throughout the spectrum. As the main cognitive theories of ASD, including the theory of mind deficit hypotheses, weak central coherence account, and the executive dysfunction theory, still fail to explain the broad spectrum of symptoms, a new perspective on the etiology of ASD is needed. Deficits in working memory are central to many theories of psychopathology, and are generally linked to frontal-lobe dysfunction. This article will review neuropsychological and (functional) brain imaging studies on working memory in adolescents with ASD. Although still disputed, it is concluded that within the working memory system specific problems of spatial working memory are often seen in adolescents with ASD. These problems increase when information is more complex and greater demands on working memory are made. Neuroimaging studies indicate a more global working memory processing or connectivity deficiency, rather than a focused deficit in the prefrontal cortex. More research is needed to relate these working memory difficulties and neuroimaging results in ASD to the behavioral difficulties as seen in individuals with a disorder on the autism spectrum.

## Review

Autism spectrum disorder (ASD) is a heterogeneous neurodevelopmental syndrome in the category of pervasive developmental disorders (*Diagnostic and Statistical Manual of Mental Disorders, 4th Edition* (DSM-IV)), with an unknown etiology. Clinicians and researchers usually include autistic disorder, Asperger syndrome, and pervasive developmental disorders not otherwise specified as subtypes of ASD. Although not part of the diagnostic classification and not formal subcategories of ASD, a distinction is also often made between low-functioning autism (LFA) and high-functioning autism (HFA). No consensus criteria regarding LFA and HFA exist, but low-functioning individuals with autism are generally considered to have an IQ below 70 or 85, whereas high-functioning individuals with autism tend to have an IQ above 70 or 85 (depending on the preferred IQ cut-off point of one or two standard deviations below mean).

Although estimates vary, the prevalence rate of ASD is approximately 6 per 1,000 children, with males being affected two (autism disorder) to four (Asperger syndrome) times more often than females [1]. Across the spectrum, ASD is by definition characterized by social impairments, communication difficulties, and repetitive interests and behavior (DSM-IV). Executive function problems and (specific) learning difficulties are also found throughout the spectrum, but are not seen as core deficits [2-7]. Traditionally, ‘executive functions’ is an umbrella term for functions such as working memory (WM), inhibition, planning, impulse control, and shifting set as well as the initiation and monitoring of action [4]. As such, executive functions are generally seen as central to human cognition [8-10] and as a consequence, executive dysfunction has a high impact on daily life.

Of these executive functions, WM and inhibition are generally considered to be core components [11,12]. Although the precise definition of WM may depend on the field of interest, for example, in human or non-human studies [13], it is generally considered to be a temporary storage system under attentional control that is the basis for our capacity for complex thought [14]. This view is supported by abundant empirical studies revealing relationships between WM and reading, mathematics [15-18], reasoning and problem-solving [19-21], and general fluid intelligence [22,23]. The processes involved in WM are threefold: active conservation of information for short periods of time, context-relevant updating of information, and rapid biasing of task-relevant cognitions and behaviors.

**Figure 1:**
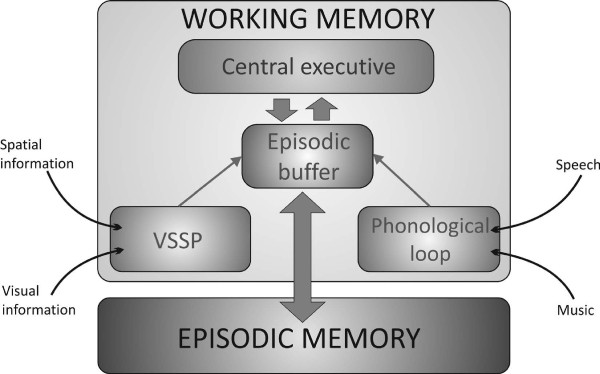
**Baddeley’s (2012) working memory model.** VSSP: visuospatial sketchpad.

Nowadays, the most commonly used cognitive model of WM is the revised WM model proposed by Baddeley [14] (Figure 1). This model contains a central executive (attention control center), an episodic buffer (which comprises a limited capacity storage system and integrates information into coherent objects and episodes) and two slave systems: one for visual and/or spatial information (the visuospatial sketchpad) and one for auditory information (the phonological loop). The model also describes links between WM, long-term memory, and perception, and has proven to be very valuable in WM studies that focus on abstract information processing, such as for numbers, locations of objects, words, and sentences [14], the so-called ‘cold’ (based on reason) cognitive processes. However, based on this revised WM model of Baddeley, we argue that WM is not only important for ‘cold’ cognitive processes (auditory and visuospatial), but is also essential for successfully navigating in the social world. Complex social situations demand online information processing of both ‘cold’ and ‘hot’ (based on motivation and emotion) cognitive information, and put a lot of stress on WM capacity [24-26]. In addition, Baddeley states that adequate social behavior can be seen as a dual task, through balancing one’s own needs and desires with those of the people with whom one is interacting, and as such requires a form of WM [27]. Although this last notion was proposed more than a decade ago, only a few studies have considered this subject, which will be discussed below.

LoPresti *et al*. [28] examined how continuously changing social information is maintained in WM. They used two versions of a delayed match-to-sample task to examine two socially salient cues: emotion and identity. Participants either had to match the emotional expression of a face to a sample face (the identity of the face was constant), or match the identity of a face to a sample face (the emotional expression was constant). Their results demonstrated sustained delay-related activation in the orbitofrontal cortex, the amygdala, and hippocampus, for both identity and emotion. These regions play a critical role in binding together emotional information in long-term memory, and are also critical for actively maintaining and binding together salient social cues in WM [29]. Other evidence for the involvement of WM in processing social information was given by Phillips and colleagues [30]. They explored the role of verbal working memory in decoding emotions and found that the process of labeling the emotions portrayed on facial expressions places high demands on WM resources. These demands increase as the number of labels that are available increases, as will be the case in real-life situations: decoding social cues from faces, bodies, and verbal material will all involve substantial investment of WM for accurate performance. The authors further mentioned that possible critical factors that might influence the WM load may be the type of decision required, the extent to which the social properties of the stimuli can be easily detected and verbalized, and the complexity of the stimuli and response requirements. Bankó *et al*. [31] found evidence that humans can store fine-grained information related to facial emotions and identity in short-term memory with high precision. Such high-fidelity short-term memory processing is crucial for the ability to monitor emotional expressions efficiently. As a result, it could be hypothesized that impairment of high-precision short-term memory storage of emotional information might (partly) underlie the deficits of emotional processing found in psychiatric disorders, including autism. Through these processes, WM not only plays a central role in processing complex cognitive information, but also an essential role in social cognition and interpersonal interactions.

During adolescence major changes take place in the neural subsystems that serve social cognition, WM, and other executive functions [32]. Adolescence is a period in which social-cognitive skills become increasingly important. To achieve adult social competence levels, adolescents have to learn how to adapt their behavior to rapidly changing social environments, in which the opinions and evaluations of peers become increasingly salient [9,33]. Both social rule use and WM are likely to develop slowly and reach maturity in adolescence or early adulthood [34-36]. The simultaneous development of both higher cognitive and socio-affective brain systems during adolescence, and the interplay of these systems on each other [37], makes this period especially interesting to study. While we acknowledge that the brain-behavior relationship of social-cognition in adolescence is complex, with many interacting processes such as changing hormone levels during puberty, as well as motivational and environmental factors, we do not focus on these specific processes in this review. Readers are referred elsewhere for excellent empirical evidence and theoretical discussions on these topics [33,36,38].

Despite the central role of WM in social cognition and in many, if not all, higher cognitive functions, the developmental tract of WM and the often reported executive function problems, few researchers have studied WM functioning in adolescents with ASD. Theory of mind deficit hypotheses, weak central coherence account, and the executive dysfunction theory all focus on certain symptoms of ASD (see Additional file 1) but fail to explain the broad spectrum of symptoms found in ASD. Also other theories have been proposed that try to explain ASD symptomatology, such as the mirror neuron theory (for a review see [39]) and the mnesic imbalance theory [40]; however, evidence for these theories is still inconclusive. A more recent hypothesis proposed by Happé and Ronald suggests that no single unitary account will be able to explain both social and non-social features of ASD. Instead they propose a fractionable triad approach, stating that, phenotypically and genetically, variations in (the triad of) social impairments, communication difficulties, and repetitive interests and behavior, are considerably independent [41,42]. Although we agree with Happé and Ronald that no unitary account will be able to explain the broad phenotype of ASD, we disagree that cognitive deficits (such as WM deficits) would not be able to explain the social side of the triad of symptoms [42]. The underlying mechanisms of this disorder are still unknown and, considering the impact of ASD on many peoples’ lives, new ways to access the cognitive and behavioral problems associated with ASD are needed. Deficits in WM and inhibition are central to many theories of developmental psychopathology [12] and earlier research strongly suggests relationships between WM capacity impairment and cognitive and social problems in adolescents with ASD, attention deficit hyperactivity disorder (ADHD), traumatic brain injury, and young adults with schizophrenia [12,24,43-45]. Bull and colleagues [46] found that executive processes, including WM, are required in both simpler and complex theory of mind tasks, underlining the role of WM in social skills. These findings may shed new light on what may be the underlying mechanisms of autism.

This review will focus on the neuropsychological results of WM studies in HFA, as well as the possible correlates with neuroimaging studies in high-functioning adolescents with ASD.

### Neuropsychological studies on working memory in high-functioning ASD individuals

Although both theory and empirical findings suggest an interrelationship between WM and social cognition, the number of studies on WM functioning in adolescents with ASD is limited and findings are inconsistent (for an overview see Table 1). Some neuropsychological studies have failed to find any significant group differences between high-functioning adolescents with autism and normal controls on WM tasks [2,11,47,48]. Nonetheless, a number of other studies clearly demonstrate impairments associated with WM and other executive functions in high-functioning adolescents with autism. Most of these impairments are found in the spatial domain of WM. Minshew and colleagues, for example, repeatedly found reduced spatial WM abilities in adolescents with autism [49-52]. Other research groups have also found spatial WM problems in adolescents with ASD [53-56].

**Table 1:** Neuropsychological studies on working memory in high-functioning adolescents with ASD

**Study**	**Age in years**	**Participant groups**	**General IQ (SD)**	**Matching criteria**	**Working memory tests (maximum load)**	**ASD impaired?**
Ozonoff and Strayer (2001) [11]	7-18	(*n* = 25) Autism	96.3 (17.8)	Age	Running memory task (2)	N
		(*n* = 15) TS	99.0 (11.4)	Gender	Spatial memory span (5)	
		(*n* = 15) TYP	107.1 (10.5)	IQ	Box search task (5)	
Geurts *et al*. (2004) [2]	6-13	(*n* = 41) HFA	98.3 (18.4)	Age	SoP (12)	N
		(*n* = 54) ADHD	99.5 (11.5)	Gender		
		(*n* = 41) TYP	111.5 (18.0)			
Goldberg *et al*. 2005 [55]	8-12	(*n* = 17) HFA	96.5 (15.9)	Age	CANTAB SWM (8)	Y
		(*n* = 21) ADHD	113.8 (10.3)			
		(*n* = 32) TYP	112.6 (12.1)			
Landa and Goldberg (2005) [54]	7-17.5	(*n* = 19) HFA	109.7 (15.8)	Age	CANTAB SWM (8)	Y
		(*n* = 19) TYP	113.4 (14.3)	Gender		
				IQ		
Williams *et al*. (2005) [52]	8-16	(*n* = 24) HFA	109.7 (16.1)	Age	WRAML FW (6)	Y
		(*n* = 44) TYP	110.0 (10.7)	IQ		
Happé *et al*. (2006) [47]	8-16	(*n* = 32) HFA, AS	99.7 (18.7)	Age	CANTAB SWM (8)	N
		(*n* = 30) ADHD, HD	99.1 (17.1)	Gender		
		(*n* = 32) TYP	106.8 (13.4)	IQ		
Verté *et al*. (2006) [12]	6-13	(*n* = 50) HFA	98.2 (17.3)	Age	SoP (12)	Y
		(*n* = 37) AS	105.2 (16.3)	Gender		
		(*n* = 25) PDD-NOS	98.3 (14.4)			
		(*n* = 47) TYP	112.1 (9.7)			
Williams *et al*. (2006) [51]	8-16	(*n* = 38) HFA	103.8 (14.3)	Age	WRAML FW (6)	Y
		(*n* = 38) TYP	107.2 (9.4)	IQ		
Luna *et al*. (2007) [50]	8-33	(*n* = 61) HFA	110.7 (16.8)	Age	ODR	Y
				Gender		
		(*n* = 61) TYP	111.0 (13.8)			
				IQ		
Steele *et al*. (2007) [49]	8-29	(*n* = 29) HFA	107.8 (11.0)	Age	CANTAB SWM (8)	Y
		(*n* = 29) TYP	110.8 (9.9)	IQ		
McGonigle-Chalmers *et al*. (2008) [57]	6.8-13.9	(*n* = 20) Autism, AS	* 76 (19.9)	Age	Size sequencing task	Y
		(*n* = 20) TYP	* 90 (8.6)			
Sinzig *et al*. (2008) [48]	6-18	(*n* = 20) HFA, AS	112 (17.7)	Gender	CANTAB SWM (8)	N
		(*n* = 20) HFA, AS + ADHD	103 (13.0)			
		(*n* = 20) ADHD	98 (13.4)			
		(*n* = 20) TYP	113 (11.9)			
Corbett *et al*. (2009) [53]	7-12	(*n* = 18) ASD	94.2(17.8)	Age	CANTAB SS (9)	Y
		(*n* = 18) ADHD	105.2(12.8)		CANTAB SWM (8)	
		(*n* = 18) TYP	112.2(14.8)			

ADHD: attention deficit hyperactivity disorder; AS: Asperger syndrome; ASD: autism spectrum disorders; FW: finger windows; HD: hyperkinetic disorder; HFA: high-functioning autism; ODR: oculomotor delayed response task; PDD-NOS: pervasive developmental disorder-not otherwise specified; SoP: self-ordered pointing task; SS: spatial span; SWM: spatial working memory; TS: Tourette syndrome; TYP: typical development. * Percentile rank.

All of the abovementioned studies used well-known and validated tasks such as the WRAML finger windows and CANTAB spatial span and spatial WM, and related the number of errors to difficulties in WM mechanisms. Two studies used different and more experimental research designs. In an oculomotor delayed response task, Luna *et al*. [50] found a developmental delay of WM in adolescents with ASD and a less refined use of WM in adults with ASD. The results indicate that WM problems are present in adolescence and despite an increase in capacity during development, adult levels of WM capacity are both reduced and delayed in ASD compared to controls. In the study by McGonigle-Chalmers [57] a size-sequencing task was used, in which participants had to sort randomly presented stars on a touch screen in either a descending or ascending order. The authors found that adolescents with ASD not only made more errors but also had higher reaction times than healthy controls. Because of the strong bias in the error data of all participants towards ‘forwards’ rather than ‘backwards’ errors, they concluded that the higher error rate and reaction times of adolescents with ASD can best be explained by deficits in the prospective component of WM responsible for the context-relevant updating of information, and rapid biasing of task-relevant cognitions and behaviors. These processes may also play important roles in the adjustment of behavior to often rapid and continuously changing social situations.

In most studies, WM problems increase when tasks impose heavier demands on working memory, for instance, when more complex or social information has to be processed [51,54,57]. In the CANTAB spatial WM task, Landa and Goldberg [54] found that children and adolescents with ASD made more between-search errors than non-ASD controls on the search task with six and eight boxes, but not on the one with four boxes. A between-search error is made when the child returns to a spatial location (box) that contains a – now irrelevant – target from a previous search. Interestingly, the number of between-search errors made by the children with ASD were related to a greater social dysfunction on the autism diagnostic observation schedule (ADOS). Similarly, Williams *et al*. [51] reported more WM problems in children and adolescents with ASD on the more complex design memory and picture memory subtest of the WRAML, whilst finding no WM problems on a less complex number/letter WM task.

These memory-load specific effects may explain why other studies did not find WM problems in adolescents with ASD. For example, the spatial span that Sally Ozonoff *et al*. used in their spatial memory-span task [11] had only a limited working-memory load. Participants had to remember the location of one, three or five colored geometric shapes over a 1,000 or 5,000 ms delay. This low task load on WM did not reveal any difficulties. In other WM studies, problems only appeared when six or eight stimuli had to be memorized [49,54]. Thus, a task using a maximum of five stimuli might simply not uncover spatial WM problems in high-functioning adolescents with ASD. Geurts and colleagues [2] failed to find an increase in working memory problems with increasing WM load on a self-ordered pointing task (SoP) in high-functioning adolescents with ASD compared with a control group. However, as they compared both groups on differences in beta weights (with difficulty level as the predictor and the number of errors as the dependent variable), no conclusions can be drawn about possible differences between the groups regarding the number of errors made on each load (6, 8, 10, or 12 items). Presumably, the stress placed on the WM system plays an important role in information-processing problems in adolescents with ASD, perhaps even more than the overall functioning of WM. The higher the information load in WM, as is the case with complex and rapidly changing social information, the more problems related to ASD emerge [12].

Other possible, but ill-studied explanations for the different results of these WM studies may lie in the compensation strategies often applied by high-functioning adolescents with ASD in structured clinical or laboratory settings. By applying compensation strategies in these highly structured settings, these teenagers often show no deficits on relatively high-level tasks for assessing the constructs of theory of mind, weak central coherence and executive functioning [58,59], while in more complex or open-ended situations (for example, social interaction) these compensatory strategies may fail. An alternative account suggests that high-functioning individuals with ASD learn the ‘rules’ of social interaction by rote (declarative memory), although they lack the intuitive flexibility (non-declarative memory) by which children with normal development ‘learn’ and apply these social rules [60-62]. This cognitive compensation strategy would demand more working memory involvement, which in turn would lead to a practice effect and a more efficient working memory network [40]. Recent neuroimaging research suggests that the transition from controlled to automatic working memory processing is associated with exponential signal decreases in task relevant regions. The temporal changes in brain activation patterns could be attributed to enhanced efficiency of information processing as a result of cognitive practice [63,64]. However, in these studies practice-related change in behavioral performance was found, for example, shorter mean response times and significantly more correct responses. This is in contrast with our findings and other studies [33]. Moreover, this effect was found interindividually within one WM task. It is not clear how these findings would translate to other WM tasks and behavior.

Thus, the complexity of the information to be processed and the stress this poses on WM seem to play a decisive role in whether or not spatial WM problems are found in high-functioning adolescents with ASD. This link between processing complex (social) information and WM is especially interesting; it gives an alternative explanation for the ‘overwhelmed feeling’ that individuals with ASD can get during complex information processing [65].

### Neuroimaging studies

Since the availability of non-invasive neuroimaging techniques, a stable and widely distributed network of cortical brain regions has been described, the fronto-parietal WM network, which is believed to be involved in WM processes in healthy adults (see Additional file 2). Until now, it has been proven to be very difficult to separate the temporal processing stages of WM and to allocate the various WM processes to different regions of the WM network. It remains unclear whether a distinction between functions cannot be found because of the inherent weakness of functional magnetic resonance imaging (fMRI) (for example, the low sensitivity and temporal resolution of the MRI BOLD signal), or because elementary WM processes are represented by a pattern of activity across the WM network. In other words, temporal processing may show the same network or different networks that are not detectable with fMRI. Nevertheless, there is a general belief that the prefrontal cortex acts as a control region in WM processes and, as one of its functions, filters out irrelevant items during the encoding of WM [66-68]. Furthermore, the dorsolateral prefrontal cortex (DLPFC) is thought to play a role in creating and maintaining links between a subject’s actions and their eventual outcomes in WM [25]. This allows previous experiences to guide the selection of future behaviors. Consistent with this view, a subject’s ability to use feedback about previous actions to guide future behaviors can be severely impaired following damage to the DLPFC. This important role of the prefrontal cortex in WM processes fits the general assumption that WM and other executive functioning problems in autism are most likely the result of some form of prefrontal dysfunction [4,5].

### Functional MRI

As stated in Additional file 2, almost the same WM network is active in adolescents as in adults, albeit more refined in the latter. Thus, although the prefrontal cortex is still maturing during adolescence, it can be hypothesized that abnormal prefrontal functioning underlies WM problems in adolescents. However, only a few published fMRI studies have examined WM processing in individuals with ASD, and only one published study has been conducted in adolescents [69] (for an overview see Table 2). In that study, high-functioning adolescents with ASD had to perform a mental rotation task in a 3-tesla magnetic resonance imaging (MRI) scanner. When their cognitive results were compared with normal controls, there were no significant differences between the ASD group and the comparison group on visuospatial task performance, response time or accuracy. Moreover, the neuroimaging results did not reveal any significant between-group differences in activation of the posterior parietal cortex. This area is thought to play a key role in mental rotation and is part of the dorsal visual processing stream that supports perception of spatial properties [70]. However, significantly less activation was found in the prefrontal area, including the anterior cingulate, dorsolateral prefrontal area, and the caudate nucleus, of the ASD participants: areas that play a significant role in the WM network in both adolescents and adults. Silk and colleagues [69] concluded that these results, despite the lack of significant differences in task performance between the two groups, may indicate a disruption of the network involved in WM and executive functions in high-functioning adolescents with ASD. These results should be interpreted with caution as the researchers did not look at the connectivity as such, and because this study was performed with a limited number of adolescents (seven adolescents with ASD and nine normal controls), especially since these results have not yet been replicated.

**Table 2:** Functional connectivity MRI studies on working memory in high-functioning adolescents with ASD

**Study**	**Mean age in years (SD)**	**Participant groups**	**General IQ (SD)**	**Working memory task (Are there differences in task performance?)**	**Results**	
					**Activation in ASD**	**Connectivity in ASD**
Silk *et al*. (2006) [69]	14.7 (2.9)	(*n* = 7) Autism, AS	114 (16.9)	Mental rotation task (accuracy: N; response time: N)	↓ Anterior cingulate, ↓ DLPFC, ↓ caudate nucleus, ↓ premotor cortex, = posterior parietal cortex	↓ Fronto-striatal
	15.0 (1.8)	(*n* = 9) TYP				
Koshino *et al*. (2008) [72]	24.5 (10.2)	(*n* = 11) HFA	104.5 (13.1)	n-back (load = 0, 1, 2) (accuracy: N; response time: N)	↓ Inferior left prefrontal region; No activation: right posterior temporal region; Shifted activation: fusiform face area	↓ Frontal-parietal
	28.7 (10.9)	(*n* = 11) TYP	108.6 (9.1)			
Solomon *et al*. (2009) [71]	15.2 (1.7)	(*n* = 22) HFA, AS	107 (14)	POP task (accuracy low trials: N; accuracy high trials: Y; response time: N)	Low control trials: =; High control trials: ↓ anterior frontal region, ↓ parietal region, ↓ occipital region	↓ Frontal-parietal
	16.0 (2.0)	(*n* = 23) TYP	113 (11)			

AS: Asperger syndrome; ASD: autism spectrum disorders; DLPFC: dorsolateral prefrontal cortex; HFA: high-functioning autism; POP: preparing-to-overcome prepotency; TYP: typical development.

This disruption in the WM network was also suggested in another study performed in a larger cohort with high-functioning adolescents with ASD. This study used a WM-related cognitive task (the preparing-to-overcome prepotency or POP task), which is thought to measure cognitive control [71]. The POP task is designed to study cognitive control involved in context processing; for example, maintaining a cue over a delay and then overcoming a prepotent response tendency. In this study, reduced activation patterns in the frontal areas, and also in parietal and occipital regions, were found in the ASD group compared to controls. The correlation methods showed reduced functional connectivity between the frontal and parietal regions in the ASD group.

Aberrant functional connectivity patterns are also found in high-functioning young adults with ASD. In an n-back WM task for faces, Koshino and colleagues [72] found a shift in activation patterns in the young adults with ASD compared with a normal control group. That is, the autism group showed less activation in the left frontal area, and no activation in the superior and middle gyri of the right posterior temporal lobes (associated with processing social information). In addition, the activation pattern in the fusiform face area was shifted towards a more lateral and inferior region. Functional connectivity results revealed that the fusiform activation in autism was less synchronized with frontal areas than in controls. Moreover, this activation pattern was not accompanied by activation in areas associated with social information processing. Despite these activation differences, cognitive performance was similar for both groups with high levels of accuracy and fast response times.

Thus, these studies show barely any differences in task performance and fMRI results revealed no overall diminished brain activation during WM tasks in ASD adolescents. Yet, the patterns of activation in the fMRI studies were different from those of normal controls. This difference in activation patterns could indicate that adolescents with ASD use a different kind of WM network to achieve the same cognitive outcomes. This may represent a compensatory mechanism to preserve cognitive functioning [69]. It is, however, unclear whether this compensatory strategy successfully works outside the structured laboratory setting in a more complex and demanding setting where WM load is even higher.

The reduced functional connectivity patterns of the WM network in adolescents with ASD seem to fit well within the cortical underconnectivity theory proposed by Just and colleagues in 2004 [73]. This theory states that individuals with autism have a reduced functional connectivity throughout the brain, resulting in a deficit in integrating information at neural and cognitive levels. These deficits are most likely to arise when the tasks require integrative processing at a high level, regardless of the domain of the task. However, the results of the abovementioned studies may also be in agreement with other connectivity theories of autism, which propose hyperconnectivity of local networks as opposed to global hypoconnectivity [74] or a combination of both hyperconnectivity of local networks and hypoconnectivity of global networks [75]; for a review see [76].

### Structural MRI

In contrast to the shortage of published research using fMRI and specifically studies on functional connectivity in adolescents with autism, much research has focused on the underlying structural brain abnormalities of autism. These structural MRI studies looked at a variety of brain characteristics such as cortical folding patterns, brain growth patterns, brain enlargement, brain volume, cortical thickness, and white-matter networks.

Earlier post-mortem and neuroimaging studies showed an abnormally high total brain volume and head circumference in young children with ASD; for a review see [77]. These findings led to a theory of age-specific anatomic abnormalities in autism, first proposed by Courchesne and colleagues in 2001 [78,79]. This theory states that there are at least three definable periods of pathological brain development in ASD. Abnormal development starts with accelerated brain overgrowth soon after birth until young childhood. This period is followed by a period of abnormally slow brain growth between young childhood and older childhood/preadolescence. Subsequently, a third period starts in adolescence (and continues into young adulthood), which is marked by a premature and arrested growth of brain size; a period when the brain growth of non-ASD adolescents catches up with that of ASD adolescents.

In the last decade support for this theory has been found by a variety of volumetric MRI studies [79-83], which did not show abnormal total brain volumes in adolescents with ASD. These results may pinpoint adolescence as a time during which the cortical growth trajectories diverge a third time, which may affect the development of cognitive and functional milestones [84-86].

While no differences were found in total brain volume compared with typically developing teens, a variety of significant regional brain differences in adolescents with ASD were found: an enlarged prefrontal cortex, enlarged right inferior parietal cortex [83], thinner left parietal and temporal cortices [82], enlarged caudate nucleus [87], and a reduced thickness of the splenium of the corpus callosum [81]. Other research showed increased folding in the frontal, parietal, and temporal lobes in both hemispheres and significant differences of cortical sulcal patterns in the superior frontal sulcus, Sylvian fissure, and inferior frontal, superior temporal and olfactory sulci in adolescents with autism [88,89]. Of these areas, several are part of the WM network and, as a consequence, these brain alterations could be related to the aberrant functional connectivity patterns found in ASD. However, it remains unclear what the clinical or cognitive significance of abnormal brain maturation in adolescents with ASD is.

### Structural connectivity

In general it is believed that abnormal functional connectivity does not necessarily imply abnormal structural connectivity, but deficient structural connectivity may underlie a lack of functional connectivity. In ASD, these structural connectivity patterns are increasingly being studied with diffusion tensor imaging, an MRI-based technique that measures the directional diffusion profile of water molecules, which reflects the axonal architecture of the brain at the micrometer level [90]. With this technique, the various white-matter tracts in the human brain can be visualized and the structural integrity and connectivity can be revealed.

In high-functioning adolescents with ASD, widespread deficits in white-matter integrity have been reported frequently (for an overview see Table 3). One study reported reduced fractional anisotropy values adjacent to brain regions that have been implicated in social cognition [91], but most studies report more globalized deficits [90,92,93]. Only one study found the specific hyper- and hypoconnectivity pattern proposed by Courchesne and colleagues, that is, an excessive disorganized and inadequately selective connectivity within the frontal lobe, and signs of hypoconnectivity and poor synchronization between the frontal cortex and other brain regions [94]. However, another study states that hypoconnectivity is global in autism, and that the short-distance tracts in the frontal, temporal, and parietal lobes are not selectively spared [93].

**Table 3:** Structural connectivity MRI studies on working memory in high-functioning adolescents with ASD

**Study**	**Mean age in years (SD)**	**Participant groups**	**General IQ (SD)**	**Method**	**Results**		
					**Location**	**Fractional anisotropy**	**Mean diffusivity**
Barnea-Goraly *et al*. (2004) [91]	14.6 (3.4)	(*n* = 7) HFA	101 (12.2)	Whole-brain voxel-based analysis	Ventromedial prefrontal cortices, anterior cingulate gyri, temporoparietal junctions	↓	NR
	13.4 (2.8)	(*n* = 9) TYP	107 (8.5)				
Cheng *et al*. (2010) [94]	13.7 (2.5)	(*n* = 25) ASD	101.6 (18.9)	Whole-brain voxel-based analysis	Right posterior limb of internal capsule	↓	NR
					Frontal lobe, right cingulate gyrus, bilateral insula, right superior temporal gyrus, bilateral middle cerebellar peduncle		
	13.5 (2.2)	(*n* = 25) TYP	109.0 (9.5)			↑	
Groen *et al*. (2011) [90]	14.4 (1.6)	(*n* = 17) ASD	98 (18)	Whole-brain voxel-based analysis	Cerebrum, cerebellum	=	↑
	15.5 (1.8)	(*n* = 25) TYP	105 (9)				
Shukla *et al*. (2011) [92]	12.6 (0.6)	(*n* = 26) Autism, AS	106.0 (3.6)	Whole-brain voxel-based analysis	Corpus callosum, anterior and posterior limbs of internal capsule, inferior longitudinal, inferior fronto-occipital, and superior longitudinal fasciculus cingulum, anterior thalamic radiation, corticospinal tract	↓	↑
	13.0 (0.6)	(*n* = 24) TYP	108.2 (2.6)				
Shukla *et al*. (2011) [93]	12.6 (0.6)	(*n* = 26) Autism, AS	106.0 (3.6)	Whole-brain voxel-based analysis	Frontal lobe	↓	↑
					Temporal lobes, parietal lobes		
	13.0 (0.6)	(*n* = 24) TYP	108.2 (2.6)			=	↑

AS: Asperger syndrome; ASD: Autism spectrum disorders; HFA: high-functioning autism; NR: not recorded; TYP: typical development.

In short, although functional and behavioral studies strongly support a WM deficiency underlying the broad spectrum of problems high-functioning adolescents with ASD have, structural imaging studies point to a more global connectivity problem in the autistic brain. Abnormal growth patterns indicate a basis for the WM problems found in adolescents with ASD, but unfortunately, there are, to our knowledge, no studies that combine behavioral, functional, and structural imaging data from the same cohort of participants. Thus, no conclusions can be drawn with respect to the clinical implications of these findings.

## Conclusions

Despite years of research and numerous studies, the cause and underlying mechanisms of ASD are still under debate. Finding a cause, or at least gaining more insight into the mechanisms behind this disorder, is still a key issue for understanding autism and for designing treatments. These findings are highly relevant, especially given the impact of this disorder on everyday functioning, and the increase in the number of individuals diagnosed with ASD.

Besides the well-known triad of problems, many people with ASD experience problems in executive functioning. Given the central role of executive functions in both higher and lower cognitive processes, problems in these functions could provide a good explanation for the symptoms seen in ASD. As WM is generally seen as a central process in many, if not all executive functions, we conclude that it is highly plausible that WM plays a leading role in the symptoms seen in ASD. To date, however, cognitive studies have failed to give conclusive evidence about the relationship between (in particular, spatial) WM functioning and the symptoms seen in high-functioning adolescents with ASD. WM has different components and one can argue that the mixed results were caused by the diversity of the tasks and the different WM mechanisms that were investigated. Nevertheless, in most studies even with different designs, WM problems increase when tasks impose heavier demands on WM. Thus the complexity of the information to be processed, rather than the specific content of the information to be processed, seems to play a decisive role in whether or not spatial WM problems are found in high-functioning adolescents with ASD.

Executive functions in general and WM processes in particular are generally linked to the functioning of the prefrontal cortex. This assumption is supported by functional MRI research, which shows a WM network containing parts of the prefrontal cortex and parietal cortex. In high-functioning adolescents with ASD, previous results seem to indicate a global WM processing or connectivity deficiency, instead of a more focused deficit limited to the prefrontal cortex. Atypical activation patterns found in both the posterior and prefrontal areas support this theory. Structural neuroimaging studies point to an even more global connectivity problem in an autistic brain.

fMRI also has limitations that should be taken in account. These limitations are mainly due to the complicated brain circuitry and functional networks that create neuronal mass activity as represented by the hemodynamic signal. This mass activity and the complicated differentiation between activation patterns imply that any results need to be interpreted with caution. Nonetheless, fMRI still represents an excellent tool, one that is currently available for gaining more insights into brain function; combining fMRI results with the results from behavioral studies should give better insights into the background, differences, and similarities of various psychological and psychiatric disorders.

In our opinion, future studies should concentrate more on combining research methods. This may give researchers and clinicians more insight into the relationships between behavior, cognition (inside and outside the laboratory), and the functional integrity of the brain. Moreover, studying behavior, cognition, and the brain in the same cohort will make it easier to interpret fMRI results and the effect of (dys)functional neural networks on everyday living and potential compensation strategies. These insights may help researchers and clinicians to develop and use better intervention techniques for this group of important pervasive developmental disorders.

## Abbreviations

ADHD: Attention deficit hyperactivity disorder; AS: Asperger syndrome; ASD: Autism spectrum disorders; DLPFC: Dorsolateral prefrontal cortex; DSM-IV: Diagnostic and Statistical Manual of Mental Disorders 4th Edition; EF: Executive function; fMRI: Functional magnetic resonance imaging; FW: Finger windows; HD: Hyperkinetic disorder; HFA: High-functioning autism; LFA: Low-functioning autism; MRI: Magnetic resonance imaging; NR: Not recorded; ODR: Oculomotor delayed response task; PDD-NOS: Pervasive developmental disorder-not otherwise specified; POP: Preparing-to-overcome prepotency; SoP: Self-ordered pointing task; SS: Spatial span; SWM: Spatial working memory; TS: Tourette syndrome; TYP: Typical development; VSSP: Visuospatial sketchpad; WM: Working memory.

## Competing interests

The authors declare that they have no competing interests.

## Authors’ contributions

EB designed the study, carried out the literature search and wrote and drafted the manuscript. GT, MH, and RK contributed to the neuropsychological literature search and in writing the manuscript. JJ, WB and PH contributed to the MRI literature search and in writing the manuscript. AA participated in its design, the literature search and coordination and participated in writing and drafting the manuscript. All authors read and approved the final manuscript.

## Supplementary Material

Additional file 1 **Main cognitive theories of ASD **[2]**-**[6]**,**[12]**,**[48,49]**,**[51]**,**[53]**,**[56]**,**[58]**,**[59]**,**[95]**-**[101]**.** Click here for file

Additional file 2 **Working memory networks in adolescence **[13]**,**[66]**,**[102]**-**[110]**.** Click here for file
