# Supplementary material for: Working memory deficits in high-functioning adolescents with autism spectrum disorders: neuropsychological and neuroimaging correlates
Source: J Neurodev Disord. 2013 Jun 4;5(1):14. doi: 10.1186/1866-1955-5-14 (PMC3674927; doi:10.1186/1866-1955-5-14)
Supplement: Additional file 1 — Main cognitive theories of ASD [2]-[6],[12],[48,49],[51],[53],[56],[58],[59],[95]-[101]. [file 1866-1955-5-14-S1.doc]

Additional file: 1 Main cognitive theories of ASD

Theory of mind deficit hypothesis [95]: This refers to the ability to impute mental states to oneself and to others [96], also called ‘mentalizing.’ This theory tries to explain the social and communicative difficulties of individuals with ASD. Earlier studies have suggested a developmental delay in theory of mind, independent of general intelligence [97]. High-functioning adolescents with ASD, however, often pass relatively high-level theory of mind tasks [58,59], which makes it hard to prove this theory for this population. Brain networks involved in mentalizing are: medial prefrontal cortex, temporoparietal junction, superior temporal sulcus, temporal poles and posterior cingulate cortex/precuneus. Additional, but less commonly activated regions: emotional networks (especially the amygdala), superior temporal lobes, and prefrontal cortex.

Weak central coherence account [98]: This refers to a processing bias for featural and local information and a relative failure to extract gist or ‘see the big picture’ in everyday life. This theory explains symptoms of ASD that the theory of mind deficit hypothesis fails to explain: the repetitive interests and behavior, obsessive desire for sameness, islets of ability, idiot savant abilities, excellent rote memory, and preoccupation with parts of objects. Recent research also showed that although high-functioning individuals with ASD have the tendency to process only local features of incoming information, they also can process this information more globally [99,100]. This makes it hard to prove the weak central coherence theory in high-functioning individuals with ASD. And although this theory has been studied often, there is still a lack of specification and consensus at the neural and cognitive levels that underlie this theory.

Executive dysfunction theory [4]: This refers to the executive function (EF) problems suffered by many individuals with ASD. Earlier studies showed clear behavioral problems in various executive function domains such as inhibition, planning, cognitive flexibility, verbal fluency, and visual working memory [2,3,6,12,48,49,51,53,56,101] in high-functioning individuals with ASD. This may explain the rigidity, repetitive interests, and the cognitive non-core symptoms of ASD. Although there is still no consensus on a specific neuropsychological profile of autism, and a few studies even failed to find any executive function problems in high-functioning individuals with ASD, most studies conclude that executive functions show a delayed developmental pattern from childhood to adolescence. Functional MRI data shows evidence for a delayed developmental trajectory for children and adolescents with HFA, which may lead to a less sophisticated and flexible use of EF in adulthood [5].
