# Supplementary material for: Working memory deficits in high-functioning adolescents with autism spectrum disorders: neuropsychological and neuroimaging correlates
Source: J Neurodev Disord. 2013 Jun 4;5(1):14. doi: 10.1186/1866-1955-5-14 (PMC3674927; doi:10.1186/1866-1955-5-14)
Supplement: Additional file 2 — Working memory networks in adolescence [13],[66],[102]-[110]. [file 1866-1955-5-14-S2.doc]

Additional file: 2 Working memory networks in adolescence

Functional imaging data shows that in adolescents and adults similar fronto-parietal WM networks are involved; cortical areas including the lateral premotor cortex, dorsal cingulate and medial premotor cortex, dorsolateral and ventrolateral prefrontal cortex, frontal poles, and medial and lateral posterior parietal cortex [13,66,102-105]. However, as WM is still developing during adolescence there are also some differences. Adolescents, for example, recruit additional regions such as the hippocampus [106], or show BOLD intensity or pattern differences in the WM network while performing a WM task. Mostly these differences are explained by refinement and further specialization of the WM network during adolescence [107–110], likely due to the maturation processes of the brain: myelination, synaptic pruning, and synapse production and proliferation. Vestegaard and colleagues [108–110] further suggested that there is a relation between white and gray matter maturation and WM performance in adolescents.

Search strategy and selection criteria

We searched PubMed for articles published between 1 January 2000, and 24 Febuary 2012, with the terms ‘working memory’, ‘adolescen*’, and ‘autism’. We included articles identified from these searches and relevant references cited in these articles. Only articles published in English were included. Studies on low-functioning individuals with autism were excluded.
